# Severe Hypothyroidism Causing Pre-Eclampsia-Like Syndrome

**DOI:** 10.1155/2012/586056

**Published:** 2012-05-29

**Authors:** Annalisa Inversetti, Audrey Serafini, Marco F. Manzoni, Anna Dolcetta Capuzzo, Luca Valsecchi, Massimo Candiani

**Affiliations:** ^1^Department of Obstetrics and Gynecology, San Raffaele Scientific Institute, Milan, Italy; ^2^Department of Endocrinology and Internal Medicine, San Raffaele Scientific Institute, Milan, Italy

## Abstract

*Objective.* Analyzing and managing pre-eclampsia-like syndrome due to severe hypothyroidism. *Methods.* Presentation of a case of severe hypothyroidism due to Hashimoto's syndrome, associated with a severe early-onset preeclampsia-like syndrome, managed in our Gynecology Department. *Results.* Severe pre-eclampsia led to miscarriage at 24 weeks of gestational age in a 42-year-old woman, although we attempted to correct hypothyroidism with increasing doses of levothyroxine and liothyronine sodium. *Conclusion.* Recognizing pre-eclampsia-like syndrome caused by overt hypothyroidism from other forms of pregnancy-induced hypertension is essential for choosing the correct treatment.

## 1. Introduction

Overt hypothyroidism (low free thyroxine hormones, elevated thyroid-stimulating hormone) has an incidence of 0.3–0.5% in pregnancy. This condition may cause severe obstetric complications, such as a preeclampsia-like syndrome, as discussed elsewhere [1, 2]. Yet, distinguishing it from other forms of pregnancy-induced hypertension is a diagnostic challenge.

## 2. Case Presentation

A 17-week pregnant 42-year-old woman, with a history of Hashimoto's thyroiditis, treated with levothyroxine 150 *μ*g/day, and homozygous mutation of methylenetetrahydrofolate reductase (MTHFR), treated with Aspirin 50 mg twice a day, folic acid, and vitamins, was referred to our hospital because of onset of severe early-onset preeclampsia, characterized by high blood pressure (180/108 mmHg), proteinuria, and headache. Nifedipine 1 g three times a day was started.

Her past obstetric history was characterized by vaginal delivery of a healthy baby and three miscarriages before the 12th week of gestational age (GA).

Due to the presence of high TSH (14.9 mU/L) and low levels of free triiodothyronine and thyroxine (resp., 1.6 pg/mL and 0.65 ng/dL), levothyroxine intake was increased from 150 *μ*g to 175 *μ*g per day. The ultrasound examination revealed high uterine arteries' Doppler waveforms resistance (average RI: 0.70).

A 24-hour urine collection revealed a protein amount of 1.575 g which increased to 6.8 g within a week. Renal and adrenal ultrasonography resulted negative. Screening tests for glomerular-based diseases were all negative. Complete screening for other autoimmune diseases resulted negative. Due to the persistent high blood pressure, alpha-metildopa was added to therapy.

TSH and proteinuria reached, respectively, 34.5 mU/L and 9.8 g/24 h, and the levels of free triiodothyronine and free thyroxine continued to decrease (1.4 pg/mL and 0.50 ng/dL). Atenolol 0.5 g/day was added to therapy. An ultrasound Doppler examination performed at 19 + 3 weeks of GA showed increased resistance in the uterine arteries (average RI: 0.77) and bilateral notches; the umbilical artery Doppler waveform presented absent diastolic flow.

Abdominal ascites and pleural bilateral effusion appeared at 20 + 4 weeks of GA, so albumin and diuretics were administered.

Persistent high blood pressure required the introduction of labetalol 25 mg three times per day; high TSH values lead to an increase in levothyroxine administration (275 *μ*g/day) and proteinuria reached a peak of 13.11 g/24 h. To reduce the fast-growing TSH levels, liothyronine sodium was added to therapy (20 *μ*g twice a day). Furthermore, the woman started to manifest oliguria, treated with fluid infusion and plasma transfusion. Nitrates were added to therapy. In the following days, proteinuria started to decrease, reaching lower levels (3.1 g/24 h).

At 23 + 6 weeks of GA, the ultrasound evaluation showed rapid deterioration of the fetal condition, compatible with fetal acidosis, which resulted in death of the fetus few hours later. Immediately after miscarriage, hydralazine (8 and 6 mL) and magnesium sulphate IV were administered. In the evening, hydralazine administration was suspended and hypertension was controlled with nifedipine (1 g twice a day).

After 3 days, therapy was adjusted with 300 *μ*g/day levothyroxine and labetalol, liothyronine was stopped. Finally, hypertension and hypothyroidism seemed to be well controlled, and the patient was discharged nine days after miscarriage.

## 3. Discussion

The association of hypothyroidism and preeclampsia is not surprising, hypothyroidism being an accepted cause of reversible hypertension both in the pregnant and in the nonpregnant population, as discussed elsewhere [3, 4]. Hypothyroidism can cause vascular smooth muscle contraction both in systemic and renal vessels, which leads to increased diastolic hypertension, peripheral vascular resistance, and decreased tissue perfusion [1, 4]. Thyroid dysfunction can be associated with proteinuria, which is known [5] to result in increased excretion of thyroxine and thyroid-binding globulins. Rare cases, have been reported [6, 7] where proteinuria is severe enough to result in losses of thyroid-binding globulins and thyroxine that cannot be compensated by the body.

Given the very early onset of hypertension and proteinuria (at 17 weeks of GA), the concurrent TSH rise, blood pressure elevation, the absence of other possible preeclampsia causes and given the known correlation between hypothyroidism and hypertension (described above), we suspected a preeclampsia-like syndrome caused by that hypothyroidism. This hypothesis was further supported by the fact that the level of proteinuria begun to decline with the normalization of TSH level, before the cessation of pregnancy itself.

In order to treat hypothyroidism-related preeclampsia-like syndrome, it is important to achieve a euthyroid state (defined by normal TSH levels) [8], if necessary by employing larger than conventional doses of levothyroxine integrated with liothyronine sodium, especially when proteinuria is a complicating factor, as demonstrated by the case we have presented.
